# Transcriptome Analysis of Selenium-Treated Porcine Alveolar Macrophages Against Lipopolysaccharide Infection

**DOI:** 10.3389/fgene.2021.645401

**Published:** 2021-03-04

**Authors:** Jia-Xuan Liu, Xin-Yu Chao, Peng Chen, Yi-Ding Wang, Tong-Jian Su, Meng Li, Ru-Yu Xu, Qiong Wu

**Affiliations:** Animal Science and Technology College, Beijing University of Agriculture, Beijing, China

**Keywords:** transcriptome (RNA-seq), selenium, porcine alveolar macrophage, lipopolysaccharide, differentially expressed genes

## Introduction

Several Gram-negative bacteria, including *Actinobacillus pleuropneumoniae* and *Haemophilus parasuis*, are responsible for respiratory diseases and cause huge economic losses to the swine industry worldwide. Lipopolysaccharide (LPS) is a cell outer membrane component of Gram-negative bacteria and serves as a major pro-inflammatory stimulus binding to pattern recognition receptor Toll-like receptor 4 (TLR4) (Ciesielska et al., 2020). LPS is ubiquitous in nature and exists in high concentrations in air pollution, soil, and organic dust. Inhalation of LPS is involved in the pathogenesis of lung inflammation (Kaelberer et al., 2020).

Alveolar macrophages (AMs) are the predominant immune cells located at the air-surface interface of alveoli. Resident AMs that arise during embryogenesis and recruited AMs that originate postnatally from circulating monocytes coexist in the inflamed lung. Once infection occurs, AMs move between alveoli to sense and phagocytose inhaled bacteria before they can induce harmful lung inflammation (Neupane et al., 2020). Meanwhile, the Gram-negative bacterial LPS binding to the TLR4 of AMs initiates multiple intracellular signaling pathways and induces the production of some pro-inflammatory cytokines, such as interleukin 1β (IL-1β) (Li et al., 2017). These pro-inflammatory cytokines induce superfluous neutrophil recruitment, leading to continuous lung inflammation and injury. The activation states of AMs are divided into classically activated (M1) and alternatively activated (M2). M1-type AMs generally induced by TLR signaling and interferon-gamma (IFN-γ) secrete pro-inflammatory cytokines, and M2-type AMs generally induced by interleukin-4 (IL-4) are anti-inflammatory and typically express the transforming growth factor-β (TGF-β) (Hussell and Bell, 2014). However, the gene reprogramming and polarization states of macrophages are also affected by stimulation intensity and tissue origin. A meta-analysis of *in vitro* differentiated macrophages showed that macrophages display distinguishing activation states even after early (2–4 h) or late (18–24 h) LPS infection (Chen et al., 2019). In M1-type AMs, increased levels of reactive oxygen species, such as hydrogen peroxide, superoxide, and hydroxyl, are implicated in DNA damage and membrane dysfunction (Riazanski et al., 2020). Therefore, the cellular antioxidant capacity of AMs is indispensable for controlling the homeostasis of intracellular oxidative stress and maintaining immune defense.

Selenium (Se) is considered as a functional element of thioredoxin reductase, glutathione peroxidase, and other Se-containing enzymes and protects against oxidative injury (Silvestrini et al., 2020). LPS infection impairs Se metabolism and leads to dysregulation of selenoprotein expression in the spleen, thymus, and lymph node of pigs (Sun et al., 2017). An animal study using a chicken model of Se deficiency has demonstrated the negative correlation between Se deficiency and inflammation-related gene expression in skeletal muscles (Wu et al., 2014). Se supplementation can attenuate inflammatory response and lung injury induced by a variety of stimuli, including virus (Liu et al., 2015), bacteria (Xu et al., 2020), and heavy metal (Ghorbel et al., 2017). It was also reported that supplementation of Se to macrophages ameliorates the pro-inflammatory response induced by LPS (Vunta et al., 2008). However, the potential molecular mechanism of the anti-inflammatory function of Se is still unclear. Transcriptome sequencing is proven to be a powerful tool to comprehensively view the immune response of porcine AMs (PAMs) to bacterial or viral infection (Kim et al., 2019; Park et al., 2020). In this study, we performed transcriptome sequencing to deepen the understanding of the mechanism of Se protecting PAMs against LPS infection.

## Materials and Methods

### Cell Culture and Treatment

The porcine lung alveolar macrophage cell line 3D4/31 (ATCC CRL-2844) was cultured in RPMI 1640 medium (Invitrogen, Carlsbad, CA, USA) supplemented with 10% heat-inactivated fetal calf serum, 100 U/ml of penicillin, 100 μg/ml of streptomycin, and 1 mM of sodium pyruvate. Confluent cell monolayers were treated under three different conditions: (i) RPMI 1640 medium alone (CON group), (ii) LPS from *Escherichia coli* O111:B4 (1 μg/ml, 3 ml) infection alone (LPS group), and (iii) pretreatment with Se as sodium selenite containing 0.1 μM for 6 h followed by LPS infection (1 μg/ml) (SeL group).

### RNA Extraction, Library Construction, and Sequencing

At 12 h after LPS infection, total RNA was extracted, and the RNA integrity number was further assessed using an RNA 6000 Nano kit (Agilent Technologies, Santa Clara, CA, USA). PCR amplification was performed to obtain the final libraries. The constructed library was quantified and pooled in the flow cell. After cBot clustering, the RNA-seq libraries were sequenced using Illumina high-throughput sequencing Novaseq 6000 platform, with a paired-end read length of 150 base pairs (bp).

### Genome Alignment and Gene Annotation

The clean reads were mapped to the pig reference genome Sscrofa11 using TopHat v2.1.1. The mapped reads were assembled into transcripts using StringTie v1.3.3b. The genes were annotated by BLAST based on the Cluster of Orthologous Groups of proteins (COG), Gene Ontology (GO), and Kyoto Encyclopedia of Genes and Genomes (KEGG) databases.

### Analysis of Gene Expression Levels and Identification of Differentially Expressed Genes

The expression of genes was calculated and normalized to fragments per kilobases per million reads (FPKM) using RSEM v1.3.1. Differentially Expressed Genes (DEGs) were identified using DESeq2 v1.24.0. The *p*-value was adjusted using Benjamini and Hochberg's (BH) approach for controlling the false discovery rate. Genes with an adjusted *p*-value < 0.05 and fold change (FC) > 1.5 were assigned as DEGs.

### Enrichment, Venn, and Protein–Protein Interaction Analysis of DEGs

GO enrichment analysis based on Fisher's exact test was carried out to specify the potential roles of DEGs using Goatools v0.6.5. The *p*-value was adjusted by BH, and GO terms with adjusted *p*-value < 0.05 were considered significantly enriched. KEGG enrichment analysis was performed to evaluate significantly enriched signal transduction or metabolic pathways using KOBAS v2.1.1. A Venn diagram was generated using the R package Venndiagram. The protein–protein interaction (PPI) analysis of DEGs was based on the Search Tool for the Retrieval of Interacting Genes/Proteins (STRING) database v11.0, and the minimum STRING score was set at 1,000. The interaction with a combined score >0.4 was considered to be significant. The protein network was visualized using NetworkX.

### Data Accession Number

The raw transcriptome data have been deposited in the US National Center for Biotechnology Information Sequence Read Archive database under accession no. SRR13277478–SRR13277486.

## Results and Discussion

### Quality Control and Transcriptome Assembly

A raw dataset consisting of 487.3 million reads (~73.6 Gbps) was yielded. After filtering low-quality reads, adaptor or ambiguous sequences, and removal of contamination, 51.9 (98.73%, the percentage of clean reads), 57.1 (98.81%), and 51.9 (98.77%) million clean reads from the CON groups, 52 (98.82%), 49.2 (98.88%), and 58.3 (99.18%) million clean reads from the LPS group, and 51.2 (99.21%), 56.3 (99.24%), and 54.7 (99.29%) million clean reads from the SeL group were retained. The average of Q30 of clean reads was >94.64%, indicating that the obtained clean reads were of high quality (Supplementary Table 1). The saturation curve of sequencing showed that the FPKM values of ~22.18% of genes from the CON, LPS, and SeL groups were expressed between 0.3 and 3.5, and that only a few of 6.53% of genes were highly expressed with an FPKM value >60. Most genes with medium or above expression level (i.e., the genes with FPKM value >3.5) were nearly saturated at 40% of the sequencing reads (ordinate value tended to 1), indicating that the sequencing quantity can cover most of the expressed genes (Supplementary Table 2; Supplementary Figure 1).

### Analysis of Gene Expression

A total of 27,576 genes were found across all samples, including 25,880 (93.85%) annotated genes and 1,696 (6.15%) unannotated novel genes. Among 63,606 transcripts identified, there were 14,158 novel transcripts, including 538 transcripts with exonic overlap with reference on the opposite strand, 666 transcripts with transfrag falling entirely within a reference intron, 10,491 transcripts with potentially novel isoform: at least one splice junction was shared with a reference transcript, 2,058 unknown transcripts, and intergenic transcripts, and 405 transcripts with generic exonic overlap with a reference transcript (Supplementary Figure 2).

A total of 964 (57.72%) novel transcripts and 12,473 (90.04%) novel genes were successfully annotated by BLAST, with 437 transcripts and 7,437 genes in the GO database, 91 and 7,664 in the KEGG database, 331 and 11,080 in the COG database, 955 and 12,451 in the NR database, 366 and 11,199 in the Swiss-Prot database, and 192 and 10,015 in the Pfam database, respectively (Supplementary Table 3). According to GO analysis, catalytic activity (259 genes) in molecular function, membrane (190 genes) and membrane part (186 genes) in the cellular component, and cellular process (122 genes) in the biological process were the most enriched ontology terms (Supplementary Figure 3A). A total of 91 novel genes were classified into 110 KEGG pathways involving 32 KEGG functional categories, mainly functioning in signal transduction, endocrine system, immune system, digestive system, translation, and environmental adaptation (Supplementary Figure 3B). The COG analysis showed that 37 novel genes were assigned into 13 COG functional categories (Supplementary Figure 3C), mainly including “intracellular trafficking, secretion, and vesicular transport” (Class U; 12 genes), “posttranslational modification, protein turnover, chaperones” (Class O; 9 genes), and “chromatin structure and dynamics” (Class B; 8 genes).

### Analysis of DEGs

In the CON relative to the LPS group (CON_LPS), a total of 223 DEGs, including 28 up-regulated and 195 down-regulated DEGs, were identified (Supplementary Figure 4A; Supplementary Table 4). The top 10 known up-regulated genes were RF00030, CTF1, CCDC103, STMN3, WIPI1, RELB, PHLDA1, FLT3, CHCHD10, EXOSC6, HSD17B10, OTUD1, PPP1R14B, YRDC, GMIP, SCLY, CEBPB, and SESN2. The top 10 down-regulated genes were VMAC, ECM2, TMOD1, C17orf78, IGSF6, COCH, NAALADL1, MILR1, GSDMC, and SV2A. Out of 58 identified DEGs in LPS relative to the SeL group (LPS_SeL), 25 DEGs were up-regulated, and 33 DEGs were down-regulated (Supplementary Figure 4B; Supplementary Table 5). Se treatment induced the expression of anti-apoptosis protein BCL-2 and antioxidant defense-related glutathione peroxidase 1 (GPX1) and selenoprotein H and P (SELENOP). Se acts as a rare amino acid selenocysteine through incorporation into selenoproteins. It was reported that Se supplementation protects against apoptosis induced by reactive oxygen species or toxic heavy metal lead in a BCL-2-dependent manner (Khera et al., 2017; Wang et al., 2018). In CON relative to the SeL group (CON_SeL), out of 252 identified DEGs, 27 DEGs were up-regulated, and 225 DEGs were down-regulated (Supplementary Figure 4C; Supplementary Table 6).

### Enrichment Analysis of DEGs

**Figure 1:**
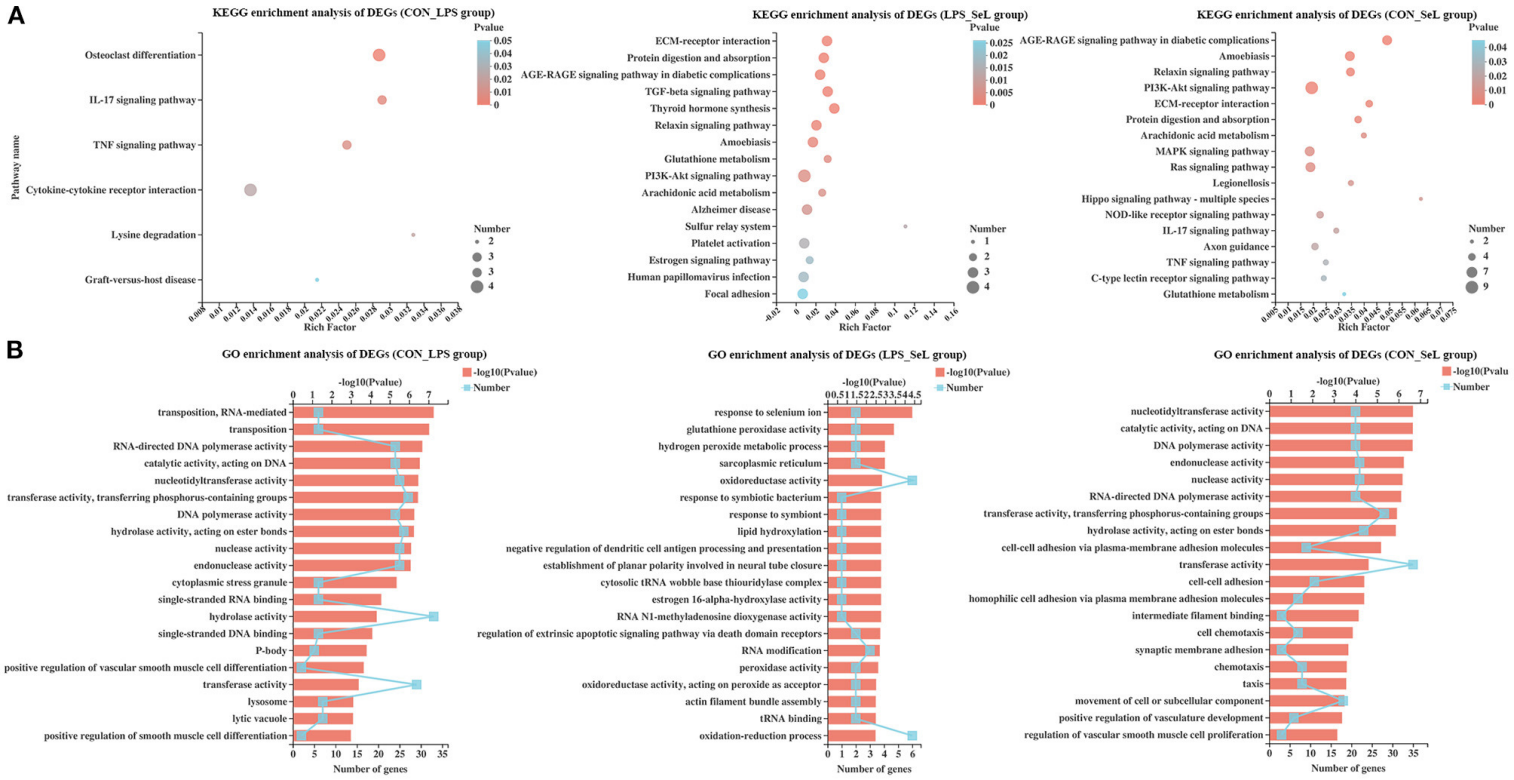
KEGG and GO enrichment analysis of DEGs. Enriched bubble chart showing the enrichment of the KEGG pathway in the CON_LPS, LPS_SeL, and CON-SeL groups **(A)**. X-axis represented the enrichment ratio, and Y-axis represented the top 20 KEGG pathways. Number: bubble size represented the number of genes annotated to a KEGG pathway. Pvalue: color indicated the enriched *p*-value. Enriched bar chart showing the enrichment of the GO pathway in the CON_LPS, LPS_SeL, and CON-SeL groups **(B)**. The length of the X-axis column represents the *p*-value. The value of the box on the fold line above X is the number of DEGs annotated to the GO terms.

KEGG enrichment analysis of DEGs was performed. The DEGs in the CON_LPS group were enriched in the IL-17 signaling pathway, tumor necrosis factor (TNF) signaling pathway, cytokine–cytokine receptor interaction, lysine degradation, and graft-versus-host disease (Figure 1A). The “TGF-β signaling pathway” possessed the highest rich factor in the up-regulated DEGs in the CON_LPS group. TGF-β could skew LPS-stimulated M1-type macrophage polarization toward the M2 phenotype *via* the Akt/FoxO1 pathway and reduce inflammatory reactions in sepsis (Liu et al., 2019). In a previous study, IFN-γ at a concentration of 50 ng/ml and LPS at a concentration of 100 ng/ml classically induce M1 activation of PAMs, accompanied by enriched TNF pathway and down-regulated TGF-β signaling pathway (Liu et al., 2018). LPS stimulation intensity could significantly affect the gene expression profile and polarization state of macrophages. Compared with short exposure (2–4 h) to LPS, short exposure (18–24 h) to LPS increases the expression of M2-related genes, including the tyrosine protein kinase MER and arginase in macrophages (Chen et al., 2019). Further study is needed to explore the regulatory effect of a high concentration of LPS on the polarization state of PAMs. Among the down-regulated DEGs in the CON_LPS group, the enriched KEGG pathways were related to “graft-versus-host disease,” followed by “endocrine resistance” and “IL-17 signaling pathway,” and “cytokine–cytokine receptor interaction” had the most DEGs. Consistent with the transcriptome analysis of PAMs activated by LPS, down-regulated genes involving cytokine–cytokine receptor interaction suggested their important role in cellular activation (Liu et al., 2018). In contrary to this study, co-infection of *Mycoplasma gallisepticum* and *E. coli* leads to inflammatory damage of chicken lung involving the enriched IL-17 signaling pathway (Wu et al., 2019). Both genes encoding matrix metallopeptidase 9 (MMP9, Log_2_FC = −1.44) and CCAAT enhancer binding protein beta (CEBPβ, Log_2_FC = 0.60) were involved in the IL-17 signaling pathway and TNF signaling pathway (Supplementary Figure 5A). The DEGs in the LPS_SeL group were highly related to categories including protein digestion and absorption, AGE–RAGE signaling pathway in diabetic complications, TGF-β signaling pathway, thyroid hormone synthesis, relaxin signaling pathway, amoebiasis, glutathione metabolism, PI3K–Akt signaling pathway, and arachidonic acid metabolism (Figure 1A). The “tryptophan metabolism” and “phototransduction” pathway possessed the highest rich factor in the up-regulated and down-regulated DEGs in the LPS_SL group, respectively. LPS and IFN-γ-stimulated RAW264.7 macrophages cultured in tryptophan-deficient medium exhibit a significant reduction in iNOS expression involved in pathogen killing (Poormasjedi-Meibod et al., 2013). The expression of proteins involved in tryptophan metabolism indoleamine 2,3-dioxygenase and kynurenic acid is activated in pig bone marrow-derived macrophages infected with LPS (Kapetanovic et al., 2012). The metabolomic analysis also showed that LPS stimulation reprograms metabolomic profiling of the human M1-type AMs and induces tryptophan degradation in tryptophan metabolism (Fall et al., 2020).

Compared with the LPS challenge, Se treatment up-regulated the expression of GPX1 (Log_2_FC = 2.12) expression and down-regulated the expression of GPX2 (Log_2_FC = −0.72), which participated in thyroid hormone synthesis, glutathione metabolism, and arachidonic acid metabolism (Supplementary Figure 5B). The presence of prostaglandin E2 (PGE2), a main arachidonic acid derivative, is necessary to the LPS-induced production of the pro-inflammatory cytokine IL-1β (Zaslona et al., 2017). Up-regulated thrombospondin 1 (THBS1, Log_2_FC = 0.64) was involved in the TGF-β signaling pathway and PI3K–Akt signaling pathway. The enriched KEGG pathways for the CON_SeL group, out of 252 DEGs, were very similar to the CON_LPS group, but ranked differently: amoebiasis, relaxin signaling pathway, PI3K–Akt signaling pathway, extracellular matrix (ECM)–receptor interaction, protein digestion and absorption, arachidonic acid metabolism, MAPK signaling pathway, Ras signaling pathway, and Legionellosis, which had many genes in common, including up-regulated genes [GPX1, TLR4, nuclear factor kappa-B (NF-κB) subunit RELβ, chemokine C-X-C motif ligand 2 (CXCL2), platelet-derived growth factor subunit B (PDGFB), GRB2-associated binding protein 1 (GAB1), etc.] and down-regulated genes [phospholipase A2 group 1β (PLA2G1β), MMP9, muscle RAS (MRAS), ephrin A2 (EFNA2), GPX2, etc.] (Figure 1A; Supplementary Figure 5C). The main limitation of this study was that the Se alone treatment group was not included, which was limited to assess the effect of Se treatment on the gene expression profile of PAMs. Se supplementation was proven to attenuate the levels of oxidative stress and pro-inflammatory gene expression in macrophages (Vunta et al., 2008; Ghorbel et al., 2017). A comprehensive gene expression profile of Se-treated PAMs is needed in further study.

GO enrichment analysis was performed. Among the DEGs in the CON_LPS group, RNA-mediated transposition and transposition classified into biological process class occupied the strongest enrichment degree. The molecular function class was the most abundant function groups, mainly including some enzyme activity, such as polymerase activity, catalytic activity, nucleotidyltransferase activity, transferase activity, hydrolase activity, nuclease activity, and endonuclease activity. The cytoplasmic stress granule was the main type in cellular component (Figure 1B). The presence of CEBPB and CD28 was directly related to the positive regulation of IL-4 production (Supplementary Figure 6A). The DEGs in the LPS_SeL group were predicted to be involved in the response to Se ion, followed by a response to the symbiotic bacterium, regulation of extrinsic apoptotic signaling pathway *via* death domain receptors, and some oxidative stress-related functional terms, including hydrogen peroxide metabolic process, glutathione peroxidase activity, oxidation–reduction process, oxidoreductase activity, peroxidase activity, and oxidoreductase activity acting on peroxide as acceptor (Figure 1B). Up-regulated GPX1 (Log_2_FC = 2.12) and SELENOP (Log_2_FC = 0.61) were related to the response to Se ion (Supplementary Figure 6B). Cytochrome P450 1A1 (CYP1A1, Log_2_FC = 2.47), GPX1, GPX2 (Log_2_FC = −0.72), and hydroxysteroid 17-beta dehydrogenase 10 (HSD17B10, Log_2_FC = −0.69) were related to oxidative stress-related function. THBS1 (Log_2_FC = 0.64) was related to the regulation of extrinsic apoptotic signaling pathway *via* death domain receptors. The DEGs in the CON_SeL group were found to be involved in the significant enrichment of GO biological terms including enzyme activity and cell adhesion function (Figure 1B; Supplementary Figure 6C).

### Functional Annotation of DEGs Affected by Both Se and LPS

**Figure 2:**
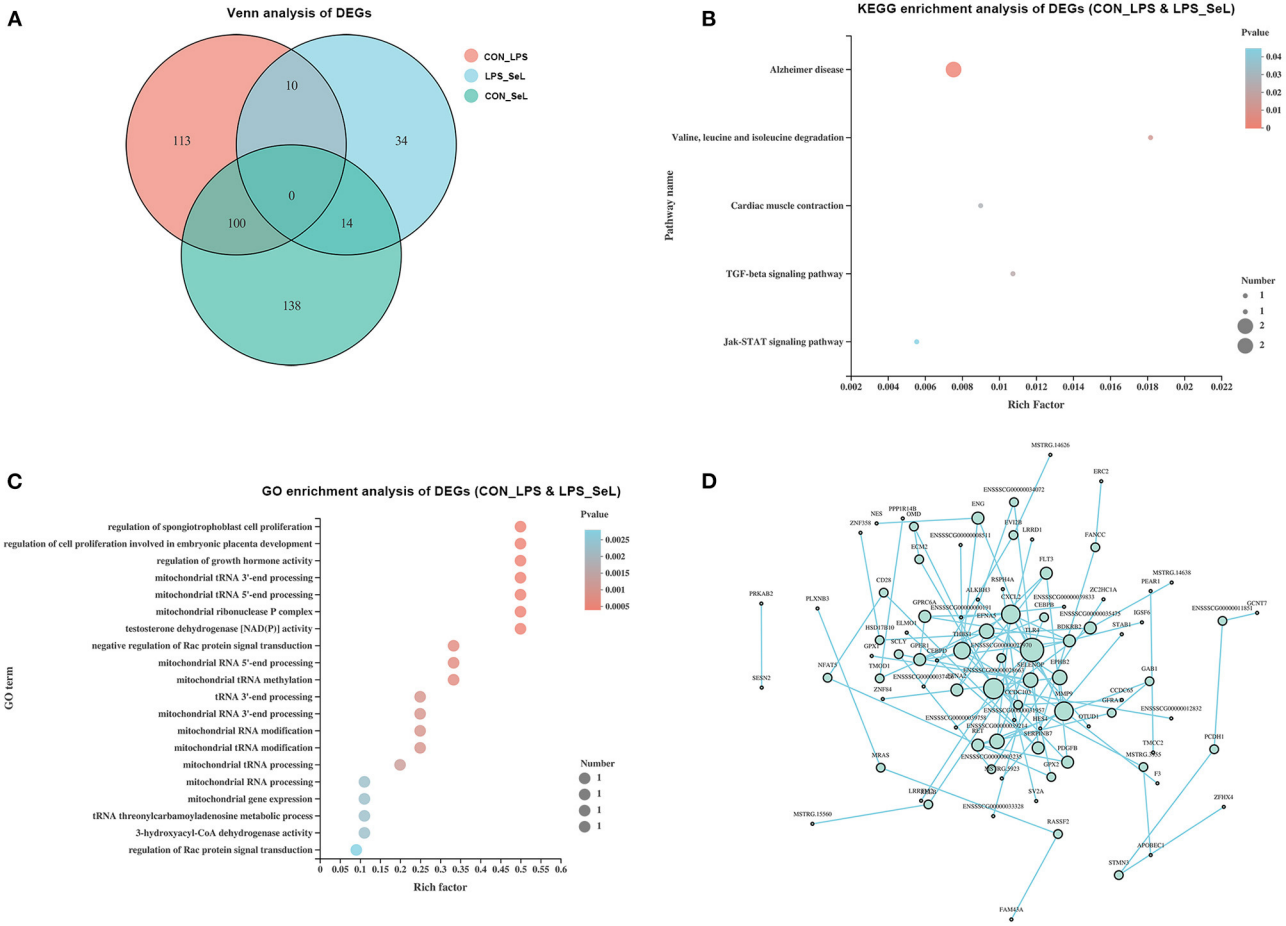
Functional annotation of DEGs affected by both Se treatment and LPS infection. Venn diagram presenting co-expressed and uniquely expressed DEGs in the CON_LPS, LPS_SeL, and CON_SeL groups **(A)**. The KEGG **(B)** and GO **(C)** enrichment analysis of DEGs affected by both Se treatment and LPS infection was indicated. PPI was performed to generate interaction analysis **(D)**. Node color from white to green represents the lowest to highest betweenness centrality. The size of each node corresponded to the number of connections (degree).

According to the Venn analysis, a total of 113, 34, and 138 genes were specifically expressed in the CON_LPS, LPS_SeL, and CON_SeL groups, respectively (Figure 2A). A total of 14 and 100 genes were shared by the LPS_SeL and CON_SeL groups and the CON_LPS and CON_SeL groups, respectively. Moreover, only 10 DEGs were shared by the CON_LPS and LPS_SeL groups. The KEGG enrichment analysis showed that the 10 DEGs shared by the CON_LPS and LPS_SeL groups were enriched in Alzheimer disease; valine, leucine, and isoleucine degradation; cardiac muscle contraction; TGF-β signaling pathway; and Jak–STAT signaling pathway (Figure 2B). Among the top 20 GO terms identified by enrichment analysis based on 10 DEGs, the first three topmost enriched were regulation of spongiotrophoblast cell proliferation, regulation of cell proliferation involving embryonic placenta development, and regulation of growth hormone activity (Figure 2C). The other GO terms were involved in mitochondrial RNA processing and modification.

### Interaction Analysis of DEGs

The main protein interaction cluster derived from 409 DEGs contained 84 nodes, each representing 1 protein and connected by 84 edges (Figure 2D). TLR4, CXCL2, MMP9, and THBS1, followed by SELENOP, had the highest scores for betweenness centrality, indicating that they accounted for many direct and indirect interactions within the network of PPI. TLR4, MMP9, CXCL2, and THBS1 had the highest number of direct connections (degree). TLR4 and CXCL2 represented the immune response of M1-type PAMs to LPS infection (Herrera-Uribe et al., 2020).

In this study, most DEGs in PAMs infected with LPS compared with the control group are enriched in the IL-17 signaling pathway, TNF signaling pathway, and cytokine–cytokine receptor interaction. LPS promotes the early activation of TLR4 and CXCL2. Se treatment enhances the antioxidant and anti-inflammatory responses to LPS through integrating GPX1, GPX2, SELENOP, CYP1A1, HSD17B10, and THBS1 genes. These findings provide an important view of the mechanism of Se protecting the host against infection. The study also suggests that dietary Se supply to pigs may help prevent respiratory infection.

## Data Availability Statement

The raw transcriptome data have been deposited in the US National Center for Biotechnology Information Sequence Read Archive database under the accession no. SRR13277478–SRR13277486.

## Author Contributions

QW and J-XL: conceived and designed the experiments and prepared the manuscript. J-XL, X-YC, PC, Y-DW, T-JS, ML, and R-YX: Performed the RNA extraction. QW, J-XL, X-YC, and PC: Performed the analysis of data. All authors contributed to the article and approved the submitted version.

## Conflict of Interest

The authors declare that the research was conducted in the absence of any commercial or financial relationships that could be construed as a potential conflict of interest.

## Supplementary Material

Supplementary Figure 1 Saturation curve of sequencing of each sample. Each color line represented the saturation curve of gene expression at different expression levels in the sample.

Supplementary Figure 2 Classification of new transcripts. New transcripts were classified according to the overlapping relationship between spliced transcripts and known transcripts. The percentage of new transcripts were shown.

Supplementary Figure 3 Annotation analysis of identified novel genes. The novel genes were annotated using based on GO **(A)**, KEGG **(B)**, and COG **(C)** databases.

Supplementary Figure 4 DEGs in PAMs in response to Se treatment or LPS infection. Volcano plots displaying DEGs in the CON_LPS **(A)**, CON-SeL **(B)**, and LPS_SeL **(C)** groups. The longitudinal dashed lines indicated an expression level of |FC| ≥ 1.5. The horizontal dashed lines indicated an expression level with a *p*-value < 0.05. Blue dots (up) represented significantly up-regulated genes; gray dots (down) represented significantly down-regulated genes; red dots (no significance) represented insignificantly DEGs.

Supplementary Figure 5 KEGGChord plot of top 20 ranked KEGG terms. Chords indicated a detailed relationship between the expression levels of DEGs (left semicircle perimeter) in the CON_LPS **(A)**, LPS_SeL **(B)**, and CON-SeL **(C)** groups and their enriched KEGG pathways (right semicircle perimeter). The genes were linked to their annotated KEGG terms via colored ribbons. Genes were ordered according to log_2_FC.

Supplementary Figure 6 GOChord plot of top 20 ranked GO terms. Chords indicated a detailed relationship between the expression levels of DEGs (left semicircle perimeter) in the CON_LPS **(A)**, LPS_SeL **(B)**, and CON-SeL **(C)** groups and their enriched KEGG pathways (right semicircle perimeter). The genes are linked to their annotated terms via colored ribbons.
