# Supplementary material for: Transcriptome Analysis of Selenium-Treated Porcine Alveolar Macrophages Against Lipopolysaccharide Infection
Source: Front Genet. 2021 Mar 4;12:645401. doi: 10.3389/fgene.2021.645401 (PMC7970123; doi:10.3389/fgene.2021.645401)
Supplement: Supplementary file 1 [file Table_1.DOCX]

**TABLE S1** Statistics of filtered transcriptome data

| Sample | Raw Reads | Clean Reads | Q30 | GC content (%) | Total mapped | Multiple mapped | Uniquely mapped |  |
| --- | --- | --- | --- | --- | --- | --- | --- | --- |
| CON1 | 52531426 | 51866502 | 94.7 | 50.5 | 49599363(95.6%) | 3309418(6.3%) | 46289945(89.3%) |  |
| CON2 | 57805816 | 57116728 | 94.8 | 50.8 | 55097556(96.5%) | 2781332(4.9%) | 52316224(91.6%) |  |
| CON3 | 52503456 | 51859562 | 94.8 | 50.8 | 50016537(96.5%) | 2555822(4.9%) | 47460715(91.6%) |  |
| LPS1 | 52584844 | 51966440 | 94.8 | 51.1 | 50118605(96.4%) | 2855115(5.4%) | 47263490(91%) |  |
| LPS2 | 49773810 | 49214958 | 95.1 | 50.9 | 47461569(96.4%) | 2417339(4.9%) | 45044230(91.5%) |  |
| LPS3 | 58801376 | 58321014 | 94.3 | 50.9 | 56264937(96.5%) | 2903919(5%) | 53361018(91.5%) |  |
| SeL1 | 51564288 | 51155636 | 94.2 | 50.9 | 49322814(96.4%) | 2444503(4.8%) | 46878311(91.6%) |  |
| SeL2 | 56685638 | 56254224 | 94.5 | 50.9 | 54229657(96.4%) | 2666746(4.7%) | 51562911(91.7%) |  |
| SeL3 | 55056494 | 54663176 | 94.6 | 51 | 52756211(96.5%) | 2567487(4.7%) | 50188724(91.8%) |  |

Clean Reads: reads of filtering out the linker and low-quality bases. Clean Base: the total number of bases filtered, ie the number of clean reads × length. Q30: bases with correct recognition rates above 99.9%. GC Content: number of G+Cs as a total number of bases percentage. Total mapped: the number of clean reads that can be mapped to the genome. Multiple mapped: the number of clean reads with multiple alignment positions on the reference sequence. Uniquely mapped: the number of clean reads with unique alignment positions on the reference sequence.
