# Supplementary material for: Transcriptome Analysis of Selenium-Treated Porcine Alveolar Macrophages Against Lipopolysaccharide Infection
Source: Front Genet. 2021 Mar 4;12:645401. doi: 10.3389/fgene.2021.645401 (PMC7970123; doi:10.3389/fgene.2021.645401)
Supplement: Supplementary file 2 [file Table_2.DOCX]

**TABLE S2** Statistics of comparing with reference genome

| FPKM Inteval | CON1 | CON2 | CON3 | LPS1 | LPS2 | LPS3 | SeL1 | SeL2 | SeL3 |
| --- | --- | --- | --- | --- | --- | --- | --- | --- | --- |
| 0-0.3 | 6022(16.60%) | 6046(16.89%) | 6064(16.93%) | 5665(15.97%) | 5825(16.39%) | 6110(17.02%) | 5879(16.48%) | 5998(16.72%) | 5912(16.57%) |
| 0.3-0.6 | 1548(4.27%) | 1514(4.23%) | 1542(4.31%) | 1436(4.05%) | 1547(4.35%) | 1559(4.34%) | 1556(4.36%) | 1554(4.33%) | 1526(4.28%) |
| 0.6-3.5 | 6509(17.94%) | 6366(17.79%) | 6404(17.88%) | 6304(17.77%) | 6525(18.36%) | 6303(17.56%) | 6379(17.88%) | 6401(17.85%) | 6431(18.02%) |
| 3.5-15 | 12855(35.43%) | 11529(32.21%) | 11437(31.93%) | 11694(31.96%) | 11385(32.03%) | 11575(32.25%) | 11560(32.40%) | 11614(32.38%) | 11547(32.36%) |
| 15-60 | 7373(20.32%) | 7936(22.17%) | 7975(22.27%) | 7985(22.51%) | 7838(22.06%) | 7971(22.21%) | 7929(22.22%) | 7954(22.18%) | 7907(22.16%) |
| >60 | 1976(5.45%) | 2402(6.71%) | 2396(6.69%) | 2392(6.74%) | 2416(6.80%) | 2372(6.61%) | 2371(6.64%) | 2345(6.54%) | 2357(6.61%) |
