# Supplementary figures and images for: Transcriptome Analysis of Selenium-Treated Porcine Alveolar Macrophages Against Lipopolysaccharide Infection
Source: Front Genet. 2021 Mar 4;12:645401. doi: 10.3389/fgene.2021.645401 (PMC7970123; doi:10.3389/fgene.2021.645401)

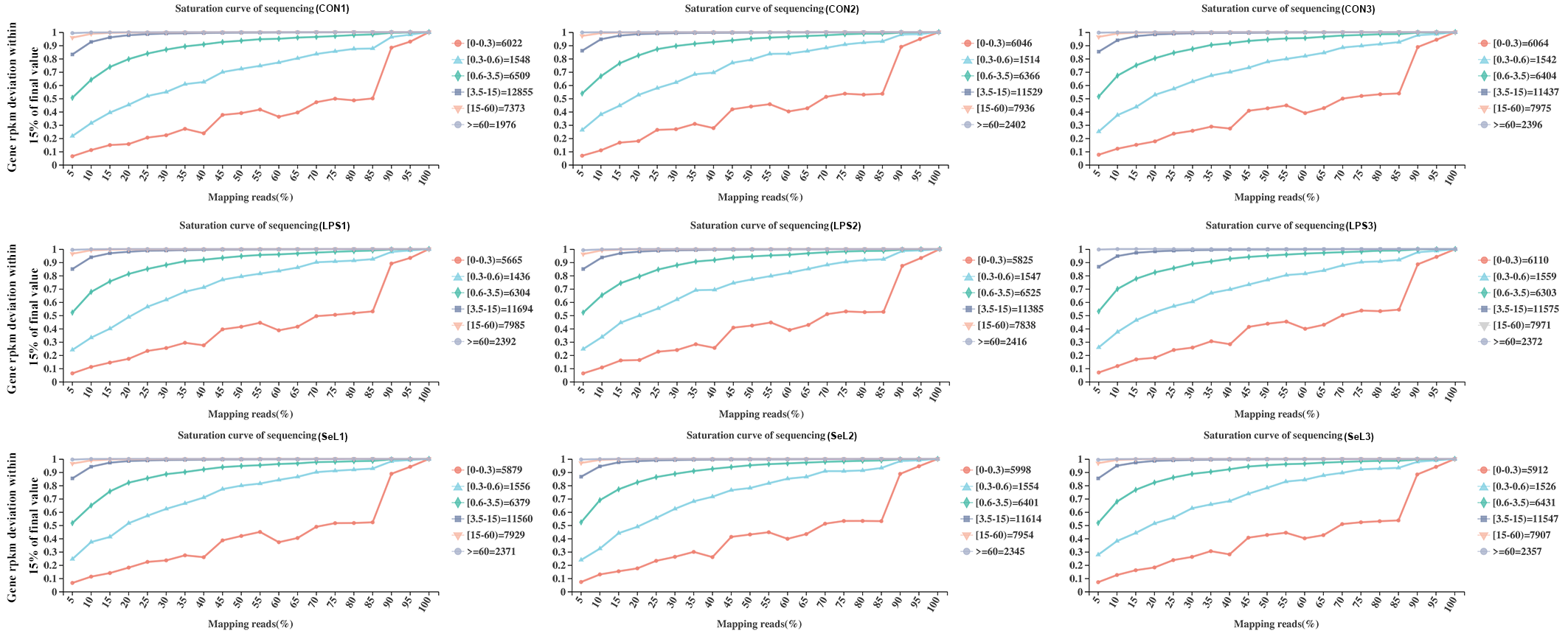

Supplement: Supplementary Figure 1 — Saturation curve of sequencing of each sample. Each color line represented the saturation curve of gene expression at different expression levels in the sample. [file Image_1.TIF]

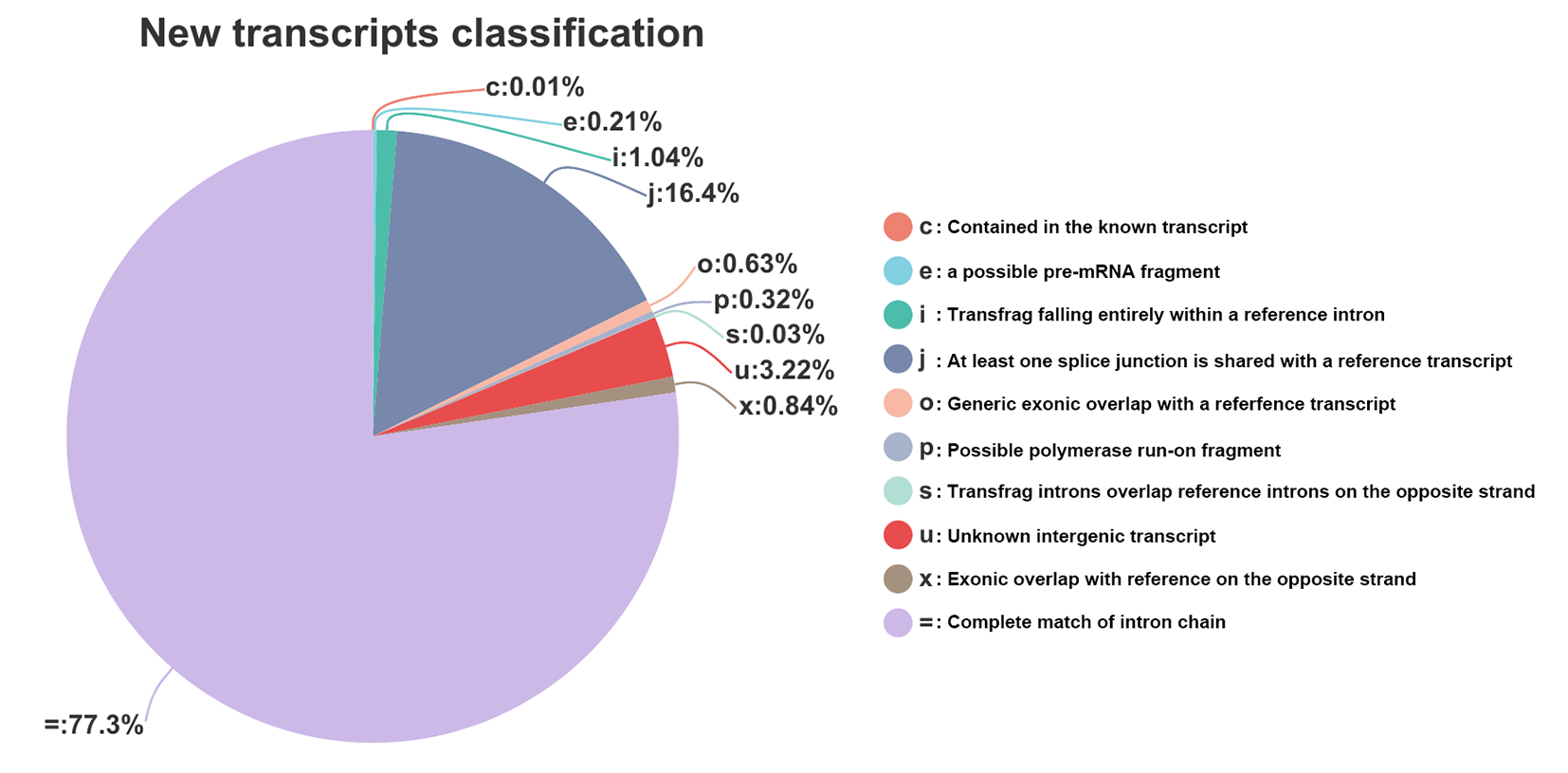

Supplement: Supplementary Figure 2 — Classification of new transcripts. New transcripts were classified according to the overlapping relationship between spliced transcripts and known transcripts. The percentage of new transcripts were shown. [file Image_2.TIF]

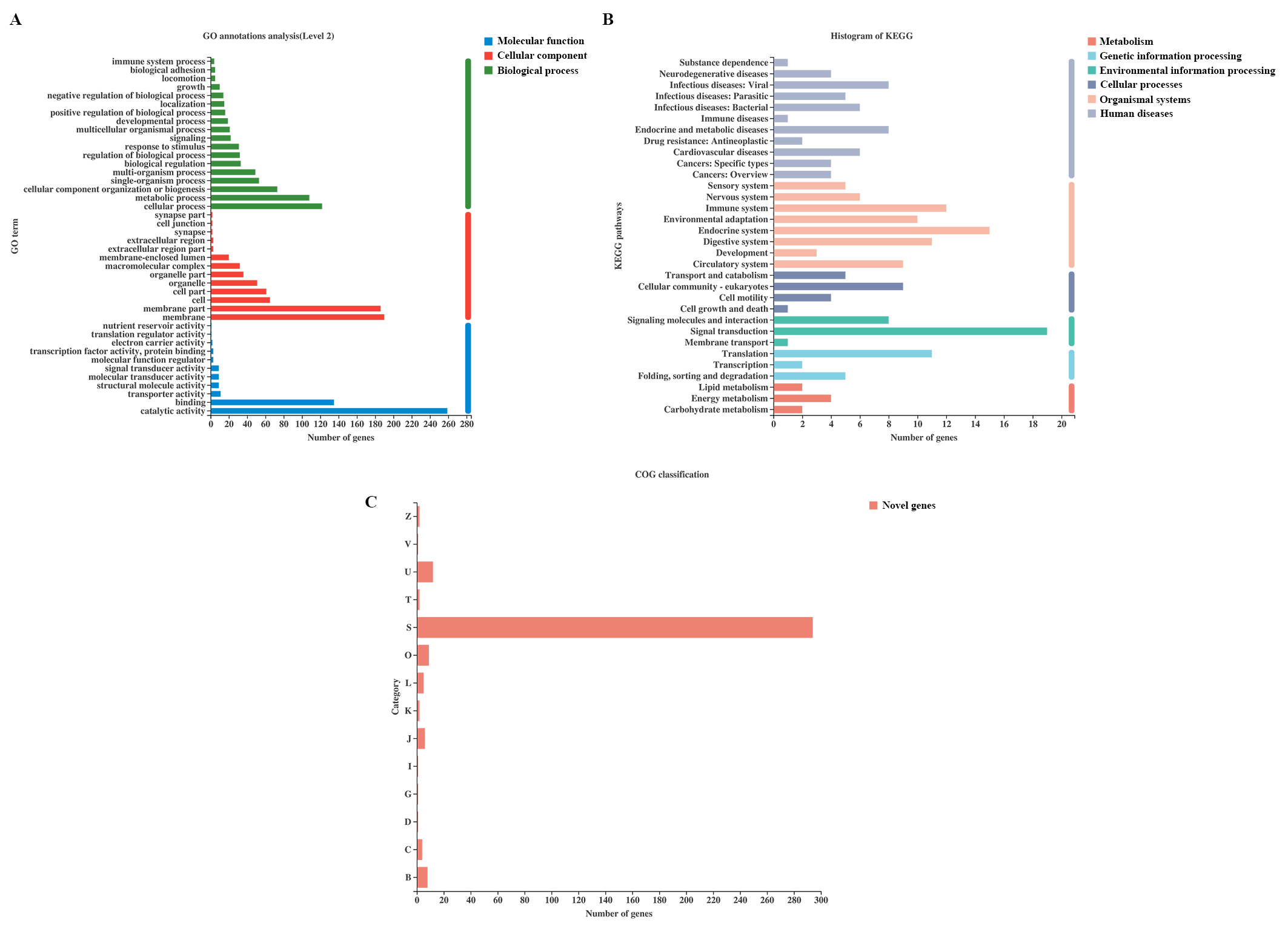

Supplement: Supplementary Figure 3 — Annotation analysis of identified novel genes. The novel genes were annotated using based on GO (A), KEGG (B), and COG (C) databases. [file Image_3.TIF]

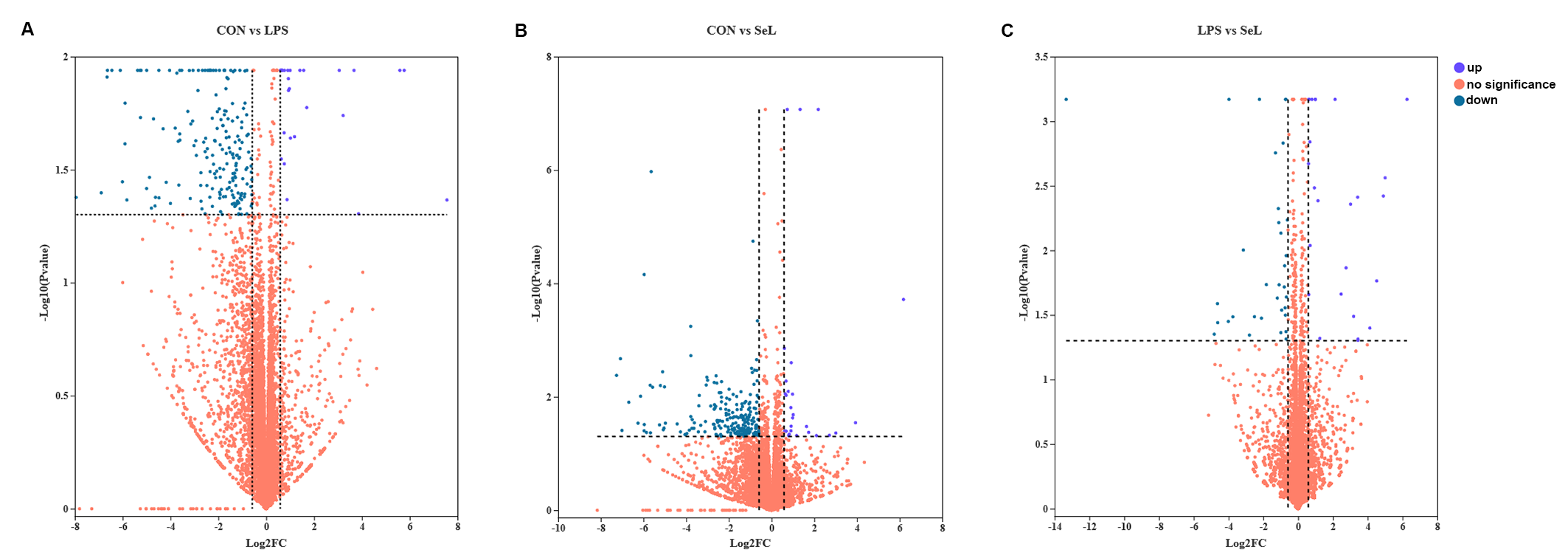

Supplement: Supplementary Figure 4 — DEGs in PAMs in response to Se treatment or LPS infection. Volcano plots displaying DEGs in the CON_LPS (A), CON-SeL (B), and LPS_SeL (C) groups. The longitudinal dashed lines indicated an expression level of |FC| ≥ 1.5. The horizontal dashed lines indicated an expression level with a p-value < 0.05. Blue dots (up) represented significantly up-regulated genes; gray dots (down) represented significantly down-regulated genes; red dots (no significance) represented insignificantly DEGs. [file Image_4.TIF]

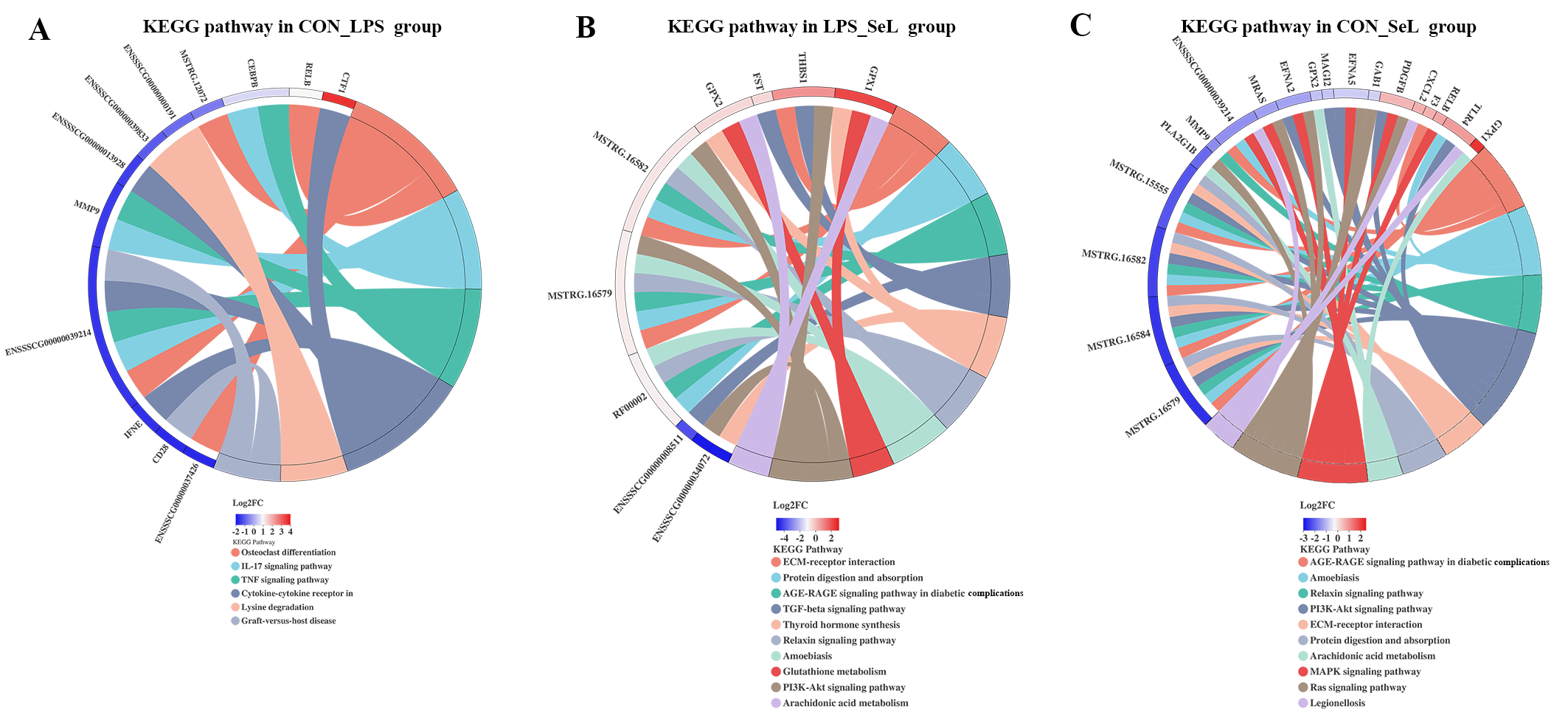

Supplement: Supplementary Figure 5 — KEGGChord plot of top 20 ranked KEGG terms. Chords indicated a detailed relationship between the expression levels of DEGs (left semicircle perimeter) in the CON_LPS (A), LPS_SeL (B), and CON-SeL (C) groups and their enriched KEGG pathways (right semicircle perimeter). The genes were linked to their annotated KEGG terms via colored ribbons. Genes were ordered according to log2FC. [file Image_5.TIF]

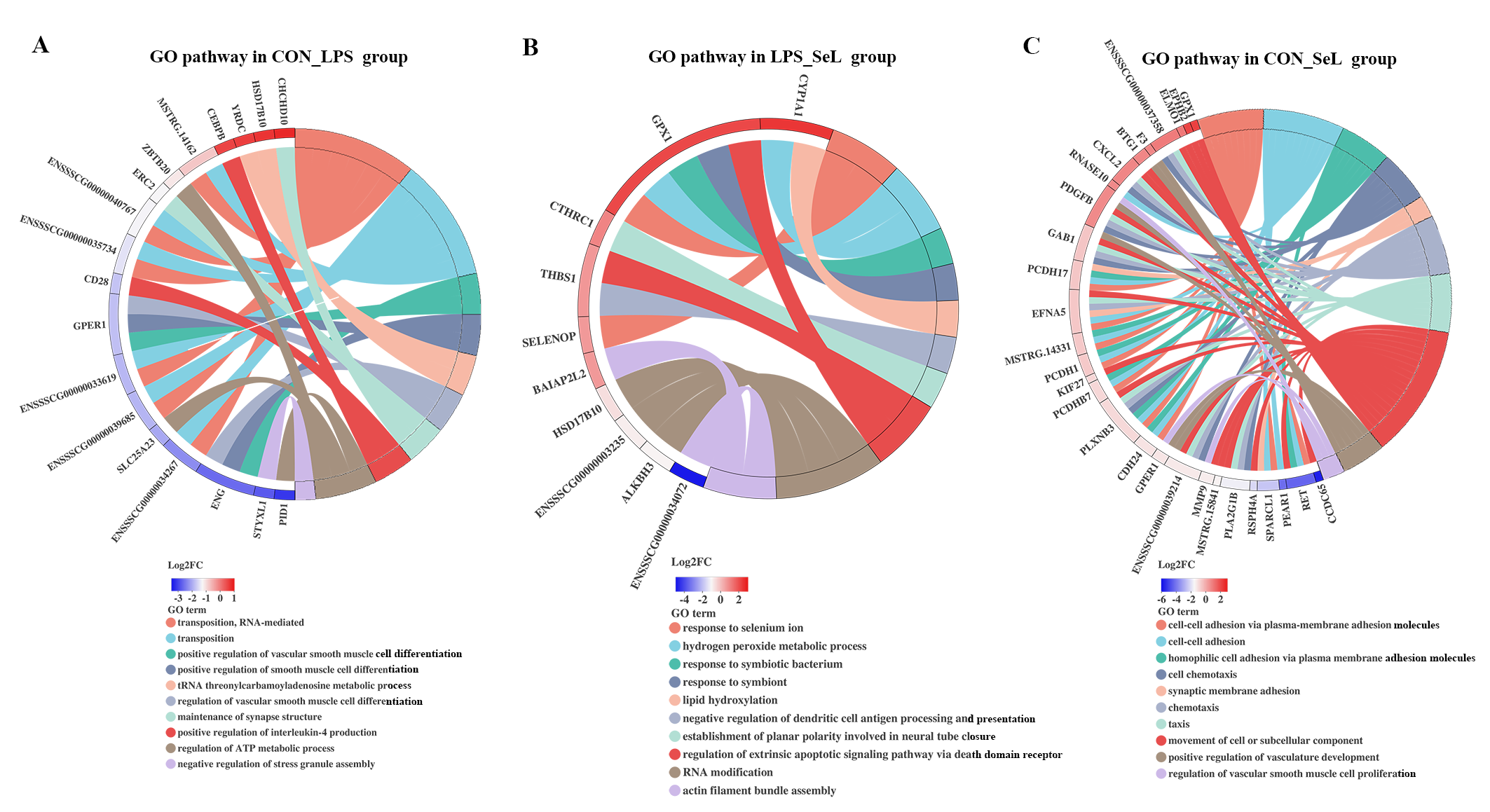

Supplement: Supplementary Figure 6 — GOChord plot of top 20 ranked GO terms. Chords indicated a detailed relationship between the expression levels of DEGs (left semicircle perimeter) in the CON_LPS (A), LPS_SeL (B), and CON-SeL (C) groups and their enriched KEGG pathways (right semicircle perimeter). The genes are linked to their annotated terms via colored ribbons. [file Image_6.TIF]
